# Retinopathy as an initial sign of hereditary immunological diseases: report of six families and challenges in eye clinic

**DOI:** 10.3389/fimmu.2023.1239886

**Published:** 2023-08-28

**Authors:** Yingwei Wang, Yi Jiang, Junwen Wang, Shiqiang Li, Xiaoyun Jia, Xueshan Xiao, Wenmin Sun, Panfeng Wang, Qingjiong Zhang

**Affiliations:** State Key Laboratory of Ophthalmology, Zhongshan Ophthalmic Center, Sun Yat-sen University, Guangdong Provincial Key Laboratory of Ophthalmology and Visual Science, Guangdong Provincial Clinical Research Center for Ocular Diseases, Guangzhou, China

**Keywords:** immunological disorders, ophthalmology, hereditary, retinitis pigmentosa, exudative vitreoretinopathy

## Abstract

**Introduction:**

Retinal degenerative or inflammatory changes may occur with hereditary immunological disorders (HID) due to variants in approximately 20 genes. This study aimed to investigate if such retinopathy may present as an initial sign of immunological disorders in eye clinic.

**Methods:**

The variants in the 20 genes were selected from in-house exome sequencing data from 10,530 individuals with different eye conditions. Potential pathogenic variants were assessed by multistep bioinformatic analysis. Pathogenic variants were defined according to the ACMG/AMP criteria and confirmed by Sanger sequencing, co-segregation analysis, and consistency with related phenotypes. Ocular clinical data were thoroughly reviewed, especially fundus changes.

**Results:**

A total of seven pathogenic variants in four of the 20 genes were detected in six probands from six families, including three with hemizygous nonsense variants p.(Q308*), p.(Q416*), and p.(R550*) in *MSN*, one with homozygous nonsense variants p.(R257*) in *AIRE*, one with compound heterozygous nonsense variants p.(R176*) and p.(T902*) in *LAMB2*, and one with a known c.1222T>C (p.W408R) heterozygous variant in *CBL*. Ocular presentation, as the initial signs of the diseases, was mainly retinopathy mimicking other forms of hereditary retinal degeneration, including exudative vitreoretinopathy in the three patients with *MSN* variants or tapetoretinal degeneration in the other three patients. Neither extraocular symptoms nor extraocular manifestations were recorded at the time of visit to our eye clinic. However, of the 19 families in the literature with retinopathy caused by variants in these four genes, only one family with an *AIRE* homozygous variant had retinopathy as an initial symptom, while the other 18 families had systemic abnormalities that preceded retinopathy.

**Discussion:**

This study, for the first time, identified six unrelated patients with retinopathy as their initial and only presenting sign of HID, contrary to the previous reports where retinopathy was the accompanying sign of systemic HID. Recognizing such phenotype of HID may facilitate the clinical care of these patients. Follow-up visits to such patients and additional studies are expected to validate and confirm our findings.

## 1 Introduction

Immunological diseases can be broadly classified into two major groups: autoimmune diseases that arise directly from dysregulated innate immune cells and auto-inflammatory diseases that result from adaptive immune responses triggered by innate immunity, both of which originated from chronic over-activation of the immune system following systemic tissue inflammation in genetically predisposed individuals (1, 2). To date, a number of immunological disorders have been identified to be hereditary, some of which have been found to be accompanied with ocular involvement that can manifest as degenerative or inflammatory changes when the retina is involved (3, 4). Retinal changes are frequently considered to be a concomitant sign of hereditary immunological disorders (HID). Despite the existence of a significant number of reported cases with HID, comprehensive descriptions regarding the accompanying retinopathy were lacking, and its distinguishing characteristics from other types of hereditary retinal diseases remained unclear. Autoimmune retinopathy was the most widely recognized retinopathy associated with HID, occurring when the eye is targeted and attacked by circulating anti-retinal antibodies of immunogenetic nature (5), in which photoreceptors, ganglion cells, and bipolar cells are predominantly affected (6). It is difficult to differentiate between autoimmune retinopathy and retinitis pigmentosa due to similar fundus performances and overlapping symptoms (7). The lack of definitive diagnostic criteria and the delayed abnormal examination indicators make significant challenges in diagnosing autoimmune retinopathy (8). Hereditary retinopathy associated with auto-inflammatory diseases has been rarely described, whereas auto-inflammatory disease has been reported to be associated to uveitis (9). In addition, there has been no comprehensive review of the genes associated with HID-related retinopathy based on a large cohort of individuals with hereditary retinal disorders. The proportion of such retinopathy, which mimics other forms of hereditary retinal diseases, within the broader spectrum of hereditary retinal disorders remains uncertain.

Here in this study, variants in 20 genes that have been reported to be responsible for retinopathy related to HID were collected from our in-house large exome sequencing data of 10,530 unrelated probands with various eye conditions (**Supplementary Table S1**). Of the 20, variants in eight were reported to cause retinal degenerative or proliferative changes, including variants in seven genes [*AIRE* (10), *ALPK1* (11), *CBL* (12), *LAMB2* (13), *MTHFD1* (14), *MVK* (15), and *TNFSF5* (16)], resulting in retinal degenerative changes and variants in *LRRC32* leading to proliferative retinopathy (17). Variants in the remaining 12 genes have been found to be associated with retinal vascular or inflammatory changes, including variants in five genes [*CECR1* (18), *IGFBP7* (19), *MEFV* (20), *NOD2* (21), and *TNFAIP3* (22)] reported to cause retinal macroaneurysms, vasculitis, or angiitis. Variants in *CAPN5* were reported to present with inflammatory vitreoretinopathy (23), hemizygous variants in *MSN* were found to cause exudative retinal detachment (24), and variants in the remaining five genes (*NLRC4*, *NLRP1*, *NLRP3*, *TNFRSF1A*, and *TYK2*) (4, 25) were associated with uveitis involving the posterior segment. All variants in the 20 genes were thoroughly analyzed by multistep bioinformatic analysis, comparison with general population gnomAD dataset, and genotype–phenotype correlation analysis. According to the American College of Medical Genetics and Genomics/Association for Molecular Pathology (ACMG/AMP) guidelines, the pathogenic variants were defined and further confirmed by Sanger sequencing and co-segregation analysis. As a result, seven pathogenic variants in four of the 20 genes were identified in six probands from six unrelated families, including six nonsense variants and one known pathogenic missense variant. The ocular recording and examination results of the six patients were reviewed in detail, and the characteristic changes on fundi were especially focused on. In contrast to previous studies on HID-related retinopathy, the distinct and novel characteristic observed in our study was that all the six patients presented with retinopathy as the initial and only sign. Based on our extensive cohort of hereditary retinal diseases from a genetic eye clinic, the genetic profile and clinical features of HID-related retinopathy were elucidated. Our findings were extremely valuable in terms of the clinical diagnosis and treatment for patients presenting with retinopathy that resemble other forms of hereditary retinopathy yet lacking defined ophthalmic genetic causes. The challenges that may arise in clinical practice were proposed, which concerned how to identify such HID-related retinopathy from an ophthalmological perspective in the absence of systemic immunological disorders and how to emphasize the significance of paying attention to these cases, which were aspired to be elucidated in future research.

## 2 Materials and methods

### 2.1 Subjects

Our study was approved by the institutional review board of Zhongshan Ophthalmic Center. A total of 10,530 unrelated probands with various ocular conditions and their available family members were recruited from our Pediatric and Genetic Clinic, Zhongshan Ophthalmic Center, Guangzhou, China. Of the 10,530 probands, 4,380 probands had hereditary retinal diseases and 6,150 individuals presented with other eye conditions. The peripheral venous blood samples of probands and available family members were collected to extract the genomic DNA using a method described previously (26), after they signed the informed consent forms adhering to the tenets of the Declaration of Helsinki. Moreover, the clinical data of each patient were carefully collected.

### 2.2 Variant’s detection and evaluation

Targeted exome sequencing, whole-exome sequencing, or whole-genome sequencing have been performed in all the 10,530 recruited probands’ genomic DNA according to a previously described method (27, 28). Variants in the 20 genes associated with HID-related retinopathy were selected from our in-house exome sequencing dataset and filtered using a comprehensive strategy outlined in our previous study (27). Variants with a low coverage depth, minor allele frequencies of ≥1% in the general population gnomAD dataset, as well as variants located in synonymous sites, untranslated regions, or non-canonical splicing sites were systematically excluded from the following analysis. All missense variants were predicted using five *in*-*silico* prediction tools, including Rare Exome Variant Ensemble Learner (REVEL; https://sites.google.com/site/revelgenomics/about), Combined Annotation-Dependent Depletion (CADD; https://cadd.gs.washington.edu), Sorting Intolerant Form Tolerant (SIFT; https://sift.jcvi.org/), Polyphen-2 (http://genetics.bwh.harvard.edu/pph2/index.shtml), and Protein Variation Effect Analyzer (PROVEAN; https://provean.jcvi.org/). The effects of variants in canonical splicing sites were assessed by the Human Splicing Finder system (HSF, https://www.genomnis.com/access-hsf). Comparative analyses were carried out between in-house dataset and the general population gnomAD database. Pathogenic or likely pathogenic variants were defined following the rigorous guidelines set by the ACMG/AMP (29), which were subsequently confirmed by Sanger sequencing, co-segregation analysis among available family members, and their consistency with relevant phenotypic characteristics (30).

### 2.3 Ocular manifestation summarization

The available clinical data of patients with pathogenic or likely pathogenic variants were collected and reviewed in detail. The recorded clinical data included gender, age of onset, age of examination, and first symptoms. Detailed ophthalmologic examinations were conducted, including best-corrected visual acuity, refractive error, fundus photography, funds autofluorescence (FAF), fluorescence fundus angiography (FFA), wide-field scanning laser ophthalmoscopy, optical coherence tomography (OCT), ocular B-ultrasound, and full-field electroretinography (ERG) according to the international standards of the International Society for Clinical Electrophysiology of Vision (ISCEV). In addition, systemic symptoms were questioned and recorded.

### 2.4 Review of retinopathy associated with immunological disorders

The term “retinopathy” and the respective gene names of the 20 identified genes were used as the keywords to search on PubMed website (https://pubmed.ncbi.nlm.nih.gov). All literature in English up to May 1, 2023 were included, and duplicates were excluded. The number of families with retinopathy attributed to variants in these 20 HID-related genes was systematically recorded and summarized. The ocular manifestations as well as systemic symptoms of these reported patients were meticulously collected and documented.

### 2.5 Statistical analysis

SPSS Statistics version 25.0 was used for statistical analysis. *Chi*-square test was used for comparative analysis of the distribution of variants and phenotypes in our in-house hereditary retinopathy cohort, in-house cohort of other ocular conditions, and the gnomAD database. *P <*0.05 was defined as statistically significant.

## 3 Results

### 3.1 Identification of pathogenic variants

**Figure 1:**
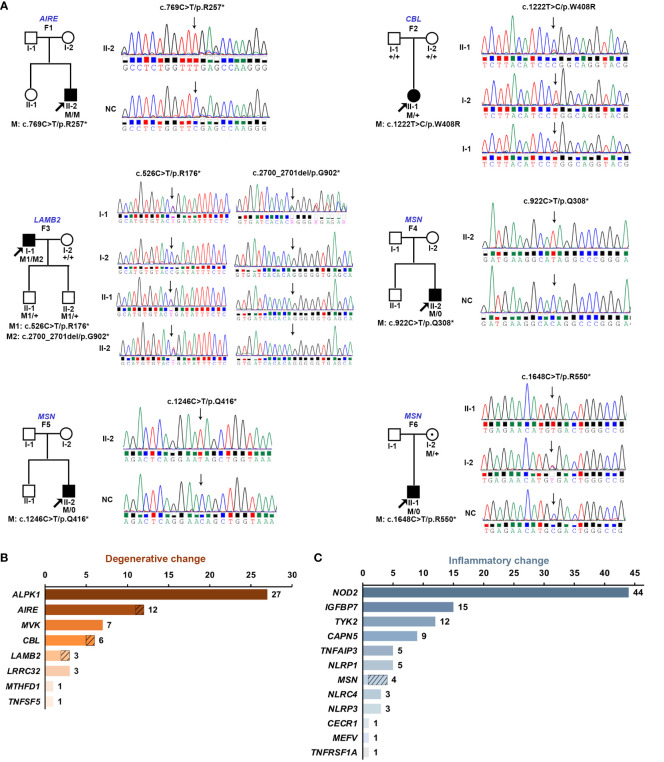
**(A)** Pedigree diagrams and Sanger sequencing consequences of six families identified with pathogenic variants in four of the 20 genes responsible for retinopathy related to hereditary immunological diseases. The squares represent male individuals, and the circles represent female individuals. The filled squares and circles indicate affected male and affected female individuals, respectively. Gene names and family numbers are provided above the pedigrees, while variants are shown below the pedigrees. The probands in each family are indicated by arrows. “M” indicates the mutant allele and “+” indicates the wild-type allele. **(B)** Genetic spectrum of eight genes associated with degenerative retinopathy including families in the literature and our cohort. **(C)** Overview of a family’s numbers with variants in the 12 genes associated with inflammatory retinopathy based on literature and our cohort. The slashed boxes in **(B, C)** represent the families identified in our cohort.

In total, seven pathogenic variants in four of the 20 genes were identified in six probands from six unrelated families, including five novels (**Table 1**; **Supplementary Table S2**). Six of the seven variants were truncation variants and were identified in five patients, including three hemizygous nonsense variants c.922C>T/p.(Q308*), c.1246C>T/p.(Q416*), and c.1648C>T/p.(R550*) in *MSN* identified in three probands, a homozygous nonsense variant c.769C>T/p.(R257*) in *AIRE* detected in one proband, and biallelic variants c.526C>T/p.(R176*) and c.2700_2701del/p.(T902*) in *LAMB2* found in one proband. The remaining one heterozygous missense variant, 1222T>C/p.(W408R) in *CBL*, was predicted as damaging by all five *in*-*silico* prediction tools and identified in one proband. Hemizygous nonsense variants in *MSN* were exclusively clustered within the in-house cohort of hereditary retinopathy, while no occurrences of these variants were recorded in the gnomAD database (*P* = 0.000009). The variant p.(R257*) in *AIRE* has been repeatedly reported in unrelated patients with autoimmune polyendocrinopathy–candidiasis–ectodermal dystrophy in homozygous or compound heterozygous status (31–33). The heterozygous missense variant p.(W408R) in *CBL* has also been reported as damaging in a prior study (34). All the seven variants in the six families were confirmed by Sanger sequencing, and those in three families were further validated with co-segregation analysis in available family members (**Figure 1A**). No other rare pathogenic variants in genes related to inherited retinal diseases were identified among all the six probands.

**Table 1:** Seven pathogenic variants of four hereditary immunological disorder-related genes identified in this study.

No.	Gene	Chr	Position	Change	Annotation	ACMG/AMP		Prediction tools						gnomAD
						Evidence	Rank	①	②	③	④	⑤	HGMD	AC
1	*AIRE*	21	45709656	c.769C>T	p.(R257*)	PVS1, PM4, PP4	P	/	/	/	/	/	DM	1 (hom)
2	*CBL*	11	119149002	c.1222T>C	p.(W408R)	PS1, PM1, PP2, PP3, PP4	P	D	D	D	D	D	DM	1
3	*LAMB2*	3	49169090	c.526C>T	p.(R176*)	PVS1, PS4, PM2, PM3, PM4, PP1, PP4	P	/	/	/	/	/	/	/
4	*LAMB2*	3	49162705	c.2700_2701del	p.(G902*)	PVS1, PM3, PM4, PP1, PP4	P	/	/	/	/	/	/	5
5	*MSN*	X	64959669	c.1648C>T	p.(R550*)	PVS1, PS4, PM2, PM4, PP1, PP4	P	/	/	/	/	/	/	/
6	*MSN*	X	64957195	c.1246C>T	p.(Q416*)	PVS1, PS4, PM2, PM4, PP4	P	/	/	/	/	/	/	/
7	*MSN*	X	64955255	c.922C>T	p.(Q308*)	PVS1, PS4, PM2, PM4, PP4	P	/	/	/	/	/	/	/

①, REVEL; ②, CADD; ③, SIFT; ④, Polyphen-2; ⑤, PROVEAN; AC, allele count; D, damaging; DM, damaging mutation.

### 3.2 Genetic landscape of immunological disorder-related retinopathy

Of the six probands with pathogenic variants detected, three had retinal degenerative changes, while the other three had retinal inflammatory changes. In addition to the three probands identified in this study, patients from 57 families with retinal degenerative disorders were reported to have variants in the eight HID-related genes associated with degenerative or proliferative retinopathy. For the 60 families with HID-related degenerative retinopathy, variants in *ALPK1*, *AIRE*, *MVK*, *CBL*, *LAMB2*, *LRRC32*, *MTHFD1*, and *TNFSF5* accounted for 45.0% (27/60), 20.0% (12), 11.7% (7), 10.0% (6), 5.0% (3), 5.0% (3), 1.7% (1), and 1.7% (1), respectively (**Figure 1B**). Conversely, except for three in-house probands with hemizygous variants in *MSN*, a total of 103 families have been reported to have variants in the 12 genes associated with retinal inflammatory changes. Therefore, variants in *NOD2*, *IGFBP7*, *TYK2*, *CAPN5*, *TNFAIP3*, *NLRP1*, *MSN*, *NLRC4*, *NLRP3*, *CECR1*, *MEFV*, and *TNFRSF1A* accounted for 42.7% (44/103), 14.6% (15), 11.7% (12), 8.7% (9), 4.9% (5), 4.9% (5), 3.9% (4), 2.9% (3), 2.9% (3), 1.0% (1), 1.0% (1), and 1.0% (1) families with retinal inflammatory changes, respectively (**Figure 1C**).

### 3.3 Ocular manifestation of six patients with HID-related retinopathy

**Figure 2:**
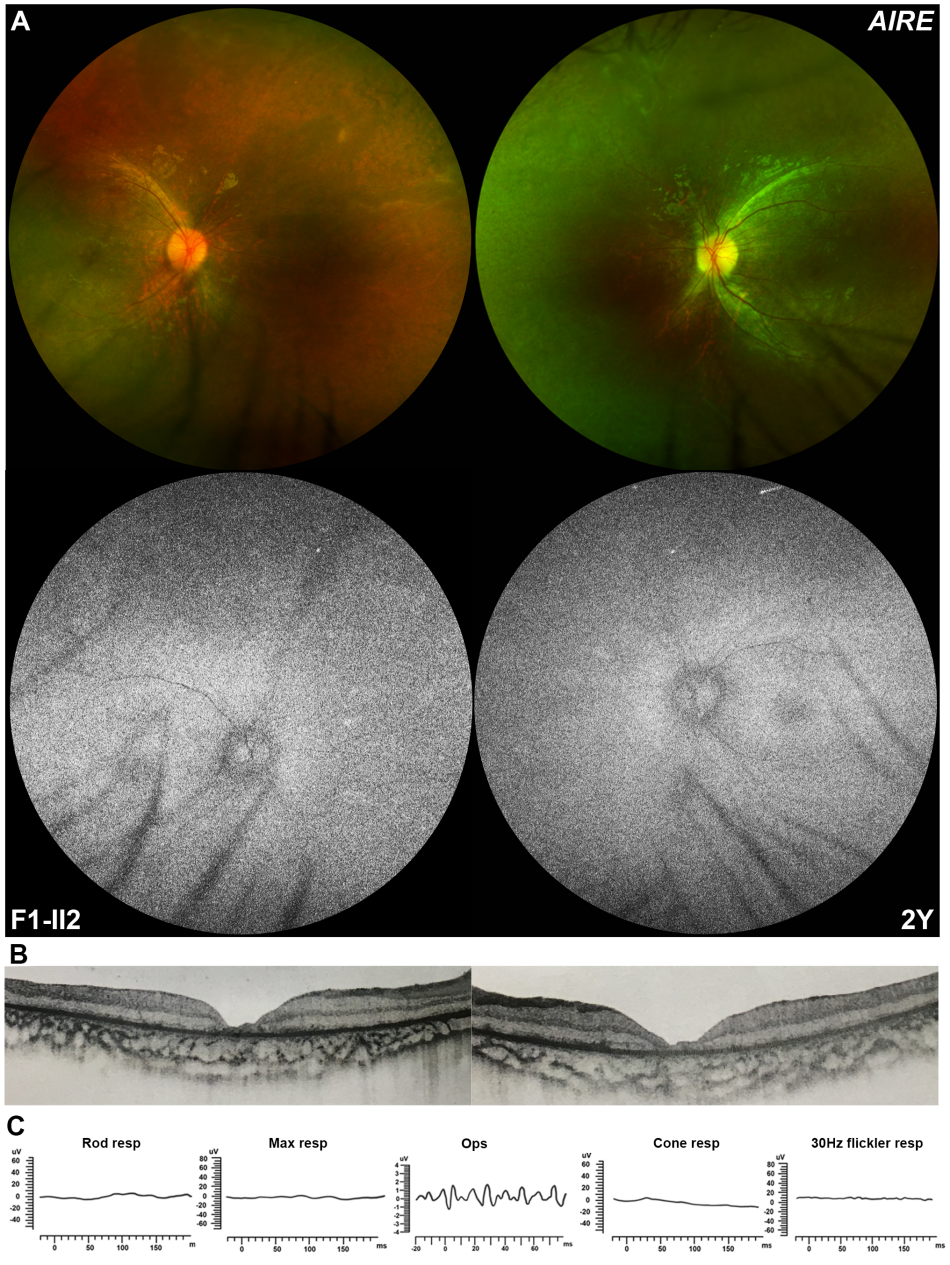
Ocular manifestation of a 4-year-old boy carrying the homozygous nonsense variant in *AIRE*. **(A)** Wide-field fundus imaging shows a pale optic disc, narrowed arterials, and obvious tapetoretinal degeneration of the whole retina without pigment deposits. Fundus autofluorescence imaging shows heterozygous hypo-autofluorescence change in the periphery and hyper-autofluorescence changes along the vascular arcade. **(B)** Optical coherence tomography showing the disruption of the outer retinal layer and ellipsoid zone of the retina, especially the macula. **(C)** Full-field electroretinography shows extinguished both rod and cone responses.

In this study, pathogenic variants in HID-related genes were exclusively detected in six of 4,380 families with hereditary retinopathy. Significantly, none of the 6,150 in-house families with other ocular conditions were detected with these pathogenic variants (*P* = 0.005). Patient F1-II2 was a 3-year-old boy identified with a homozygous c.769C>T/p.(R257*) variant in *AIRE*, whose main complaint at initial visit in our clinic was poor vision in the recent 3 months as noticed by his parents. He was unable to maintain his gaze. No obvious abnormalities were observed in the anterior segment of the eyes. Direct fundoscopy showed a waxy pallor disc and mottled change of retina in both eyes. The results of wide-field fundoscopy presented with a waxy disc pallor, attenuation of retinal arterioles, and widespread tapetoretinal degeneration in mid-peripheral retina, without bone-spicule pigmentation deposits. On FAF fundus image, heterogeneous hypo-autofluorescence changes were found in the mid-peripheral retina, with hyper-autofluorescence along the major vascular arcade (**Figure 2A**). The OCT examination demonstrated an obviously disruptive structure of the outer retinal layer and diffuse thinning of the whole retina, especially the central macula (**Figure 2B**). Both rod and cone responses were non-recordable on ERG recordings (**Figure 2C**). Cranial and orbital computed tomography showed no significant abnormalities in the cranium, both orbits, and orbital contents. The boy’s weight was slightly below the normal range for a 3-year-old boy. No other systemic symptoms were found at this visit.

**Figure 3:**
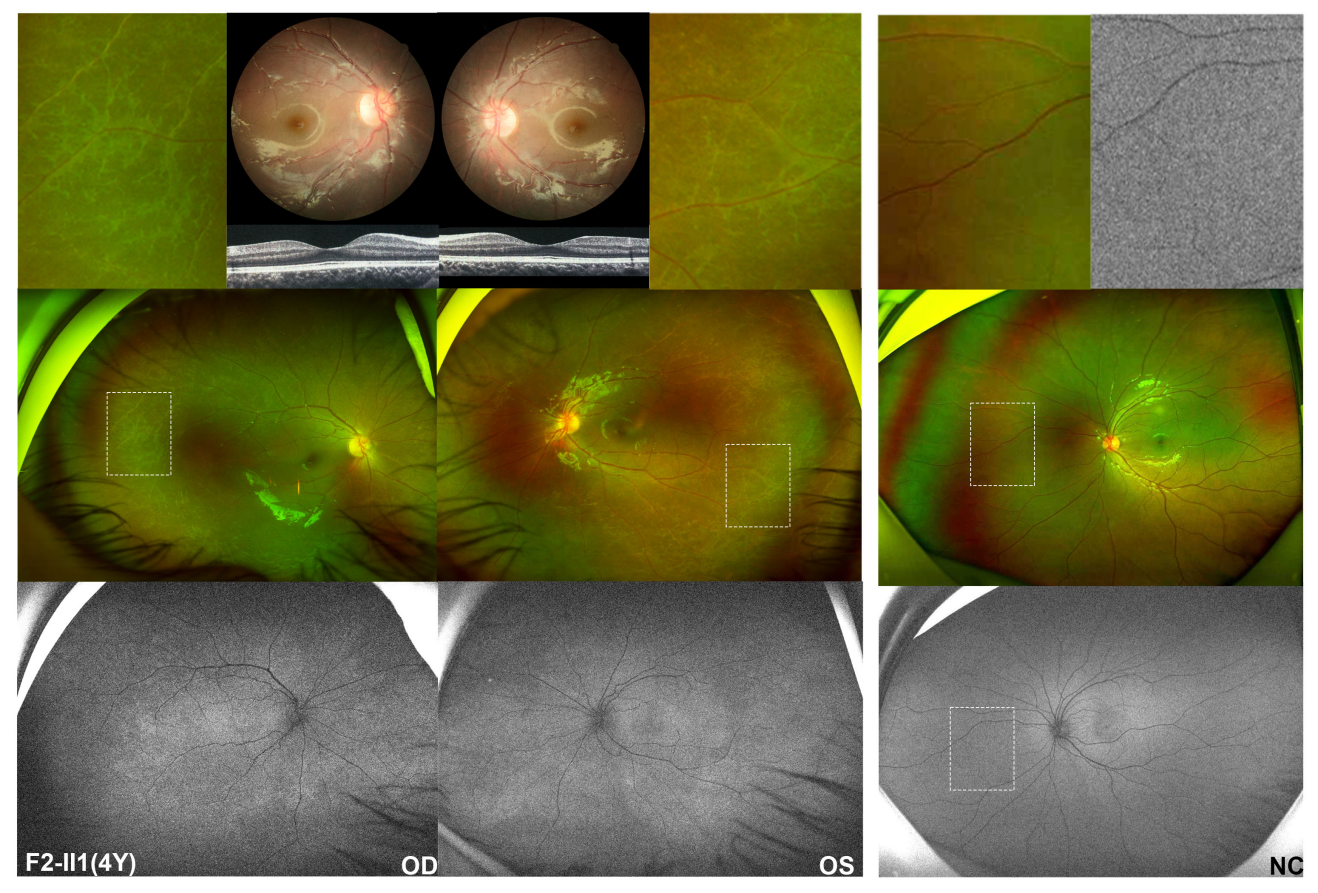
Fundus performances of a four-year-old girl identified with heterozygous pathogenic missense variant in *CBL*. The middle of the left column is the wide-field fundus photography of the patient’s right and left eye, respectively, showing slightly tapetoretinal degeneration in the mid-peripheral retina and characteristic rhagadiforme-like degeneration in the far-peripheral region, with magnified details of the peripheral changes shown above her wide-field images. The lower left imaging is autofluorescence imaging of this patient, in which speckled hyper-autofluorescence changes of the whole retina are observed, particularly evident in the macula pigmentary changes area and peripheral degenerative area. However, by routine posterior fundus examination, only macular pigmentation disorders were found in both eyes, demonstrated in the upper left. Normal macular structure was found on optical coherence tomography. The right is the left eye of a normal control, which shows no degenerative changes in the peripheral retina and homogeneous autofluorescence of the whole retina. Above the right column is a magnified view of the peripheral region in the normal control. The white dashed box indicates enlarged retinal area.

Patient F2-II1 was a 4-year-old girl with a heterozygous *CBL* pathogenic variant and who was found to have poor vision during regular physical examination at the age of three. Her BCVA was 0.6 for the right eye and 0.5 for the left, with equivalent spherical of +3.25 diopter (D) for both eyes. The anterior segment was normal. The results of a direct ophathmoscope examination revealed mild irregular pigmentary changes in the central macula and slightly tapetoretinal degeneration in the mid-peripheral retina in both eyes. On wide-field fundus examination, rhagadiforme-like degeneration was observed on the far-peripheral retina. Wide-field FAF revealed mottled changes across the whole retina, which were extremely obvious in the central macula and the far-periphery retina. The results of OCT examination showed a normal macular structure (**Figure 3**). The remaining proband F3-II1 had degenerative retinopathy and biallelic truncation variants in *LAMB2* and came to our clinic at age of 59 with a complaint of poor vision since childhood. Now, his BCVA was hand movement and finger counting for the right and the left eye, respectively. The ocular examination revealed horizontal nystagmus, partial lenticular opacity, and grayish retina with pigmentation deposits.

**Figure 4:**
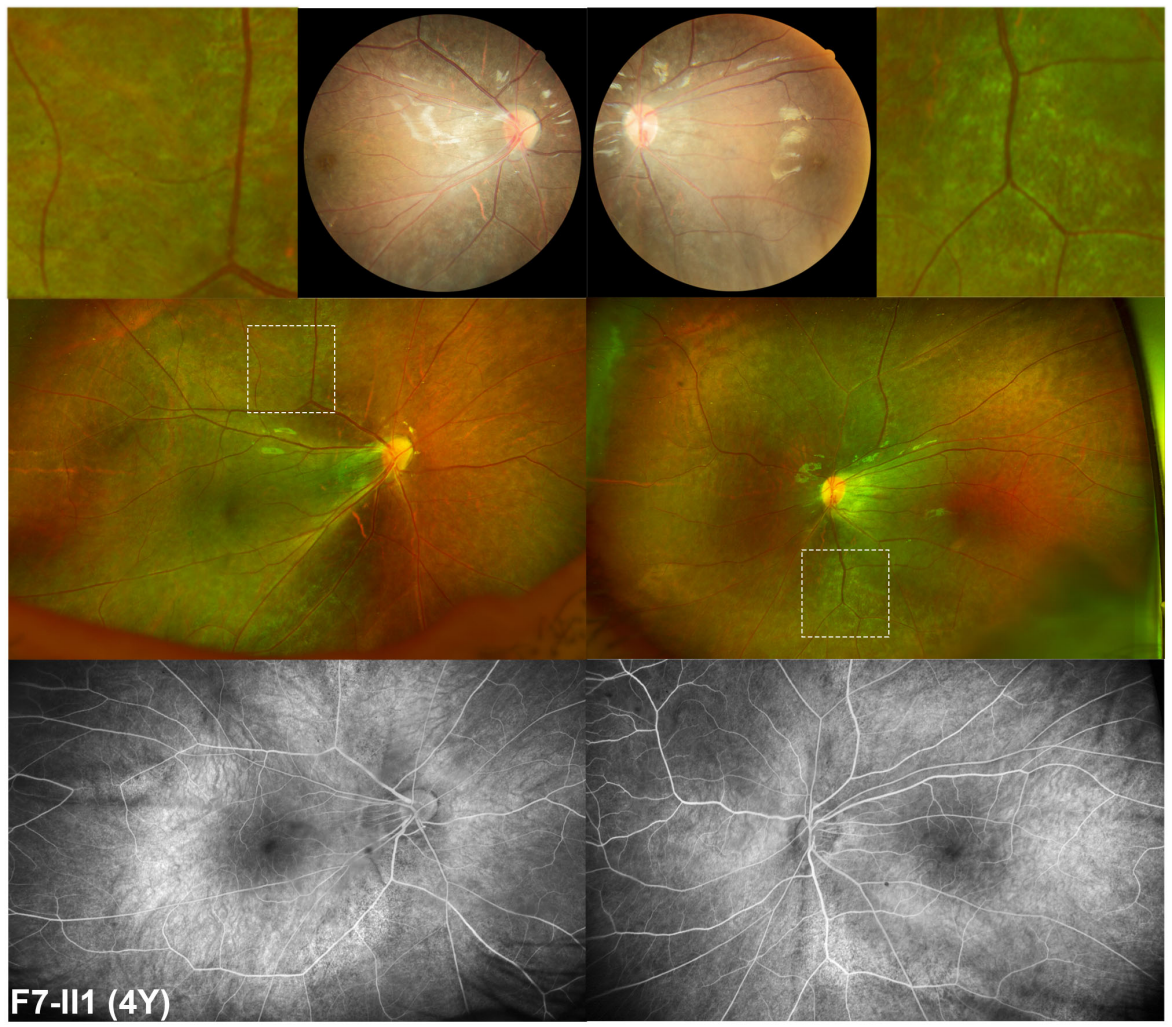
Fundus manifestation of a four-year-old boy with hemizygous nonsense variant in *MSN*. Apparent straightened of the vessels with optic vessels extending to the periphery in a fan-shape fashion and tapetoretinal degeneration of the retina were observed by posterior and wide-field fundus examination. This degeneration is different from other forms of degeneration observed in patients with retinitis pigmentosa. It is shown enlarged on the top of this image, adjacent to the posterior retina imaging. On fluorescence fundus angiography, extremely straightened retinal vessels without obvious leakage and mottled hyper-fluorescence changes around the arch of vessels are observed.

Except for the three probands with degenerative retinal changes, the other three probands with hemizygous nonsense variants in *MSN* presented with retinal changes similar to familial exudative vitreoretinopathy (FEVR) accompanied with tapetoretinal change. F4-II2 came to our clinic at the age of 6 months due to being unable to maintain his gaze as noticed by his parents. The slit lamp examination revealed a shallow anterior chamber, posterior synechia, and irregularly shaped pupil on both of his eyes. Upon RetCam examination, it was observed that the optic disc was dragged with fibrovascular membrane toward the temporal peripheral regions in both eyes. A diagnosis of FEVR was proposed, and laser photocoagulation on the retina was conducted. At follow-up visit at 10 months later, temporal tractional retinal detachment and severe temporal retinal fold were observed in the right and left eye, respectively. F5-II2 came to our clinic for decreased vision of the left eye for 1 month at the age of 13. He had phacoemulsification surgery, and an intraocular lens was implanted in his right eye due to a complicated cataract of the right eye. At that time, he had cloudy vitreous body and an intraocular membrane-like object in his right eye on B-ultrasound scan, widespread tapetoretinal degeneration and stretching of retinal vessels on posterior retina, and brush-like extraretinal neovascularization with slight fluorescein leakage in peripheral retina and far-peripheral avascular zone as demonstrated by FFA. At current visit, BCVA was 0.06 of the right eye and finger counting of the left eye. The ophthalmic examination found rhegmatogenous retinal detachment, choroidal detachment, and complicated cataract of the left eye. F6-II1 had poor vision since birth and received a series of ocular as well as systematic examination at the age of 2. White calcified opacity was found at the posterior capsule of the left eye’s lens, which suggested a diagnosis of congenital cataract. Ocular examinations were performed under general anesthesia. Vitreous haze and posterior detachment of the retina in both eyes were observed by B-ultrasound. The axial lengths were 23.79 and 23.19 mm of the right and left eye, respectively. The optometry results were -10.00DS for the right eye and -9.50DS for the left eye. The results of the fundus examination was suggestive of macular hypoplasia. At the age of 4 years, BCVA of the right eye was 0.16 and that of the left one was 0.1. Direct fundoscopy observed the retina to be slightly showing tapetoretinal degeneration, macular hypoplasia, apparent straightening of the vessels, and optic vessels extending to the periphery in a fan-shaped appearance. Wide-field fundus examination including FFA showed generalized retinal degeneration and extremely straightened retinal vessels without obvious leakage (**Figure 4**). The OCT results showed hypoplastic fovea (**Figure 4**).

### 3.4 Retinopathy features of the four genes in the literature

In a previous study, 12 patients from 11 unrelated families have been identified with homozygous or compound heterozygous variants in *AIRE* and observed with autoimmune retinopathy (10, 35–40). In total, 11 of the 12 patients initially had systemic symptoms, including autoimmune hepatitis, mucocutaneous candidiasis, hypoparathyroidism, or adrenal insufficiency, followed by vision loss or nyctalopia. Ocular manifestations resembling retinitis pigments were observed in the 11 patients who showed a pale optic disc, attenuated retinal arterioles, and bone-spicule pigment deposits in the peripheral retina. There were six patients who had nondetectable rod and cone responses on ERG and disruption of the photoreceptors layers on OCT. Their BCVA ranged from 0.1 to 1.0. Their anterior segments had no remarkable findings. The remaining one of the 12 patients was a 19-month-old patient who had a rapid decrease in vision after an infection of COVID-19 and non-recordable responses of rod and cone on ERG examination. Due to the fact that no other systemic symptoms were identified at visit, Leber’s congenital amaurosis genes were suspected. Variants in genes related to Leber’s congenital amaurosis were comprehensively screened, and no positive findings could be identified. After autoimmune hepatitis occurred at the age of 3, autoimmune-related retinopathy was considered, and pathogenic variants in *AIRE* were identified (10). Five unrelated probands have been identified with heterozygous missense variants in *CBL* and diagnosed with RASopathy accompanied with retina involvement. Their ocular manifestation was variable, including two with periphery pigmentary changes, two with optic edema or atrophy, and one with retinal detachment due to episodes of chronic uveitis (12). Two reported patients with Pierson syndrome caused by *LAMB2* variants exhibited distinct retinal manifestations, including one with retinal atrophy change and one with retinal detachment, following early-onset renal failure (13, 41). Only one published case has been observed with exudative retinal detachment following a recurrent infection history in his early life and detected with hemigynous variant in *MSN* (42). As for the remaining 39 published families identified with variants in the remaining five HID-related genes (*ALPK1*, *LRRC32*, *MTHFD1*, *MVK*, and *TNFSF5*) that have been associated with degenerative change of retina, only two probands were detected with *MVK* variants and presented with non-syndromic retinitis pigmentosa as initial symptom, while patients in the other 37 families had ocular symptoms as concomitant signs of systemic abnormities.

## 4 Discussion

**Figure 5:**
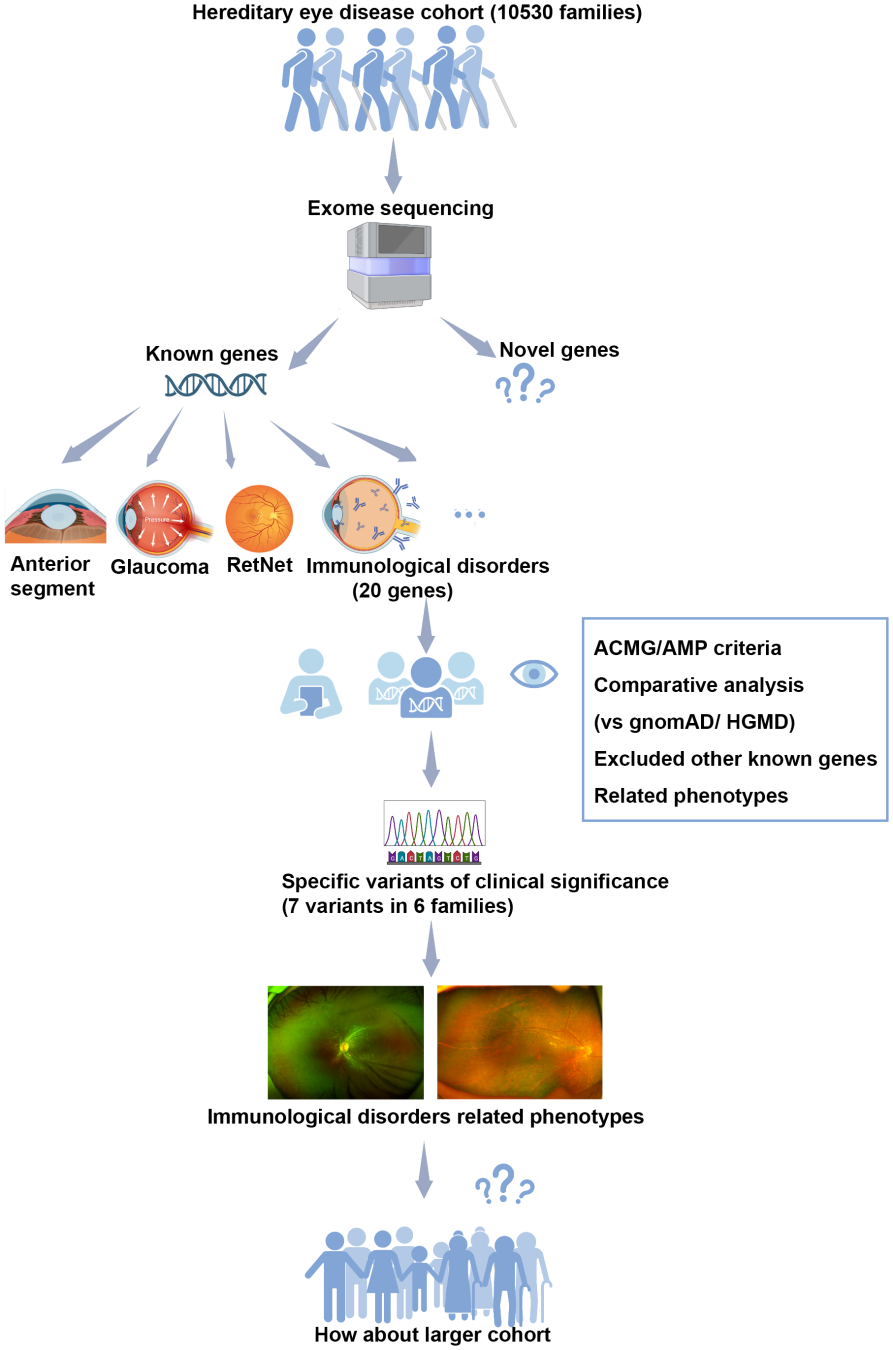
The flow diagram illustrating our analysis process in this study. Our study was conducted based on a large cohort of hereditary eye disease, comprising 10,530 probands with various ocular conditions. Exome sequencing was performed on the genomic DNA of all 10,530 families. Variants in 20 genes known to be associated with hereditary immunological disorders-related retinopathy were selected for further multistep analysis. Pathogenic variants were assessed according to the ACMG/AMP criteria, involving comparative analysis with the general population gnomAD database, reference to the HGMD database, and consideration of consistency with related phenotypes. No other variants in known genes responsible for inherited retinal disorders were identified. As results, seven pathogenic variants in four of the 20 genes were identified in six unrelated families. Retinopathy, resembling other forms of inherited retinal diseases, was observed as the initial sign in these cases. Our finding prompts the need for further observation in a larger cohort.

This study was the first research that investigated the genetic profile and clinical characteristics of retinopathy associated with HID from the perspective of a large cohort of hereditary retinal diseases, based on our in-house exome sequencing database of inherited ocular diseases. Variants in 20 genes reported to be responsible for HID-related retinopathy were collected and comprehensively evaluated. Seven pathogenic variants in four of the 20 genes were identified, whose pathogenicity was well defined, as confirmed by co-segregation analysis, compared with the general population database and referenced with published literature. Of the seven pathogenic variants, three hemizygous nonsense variants in *MSN* were exclusively aggregated in our inherited eye diseases cohort but absent in the gnomAD database. These pathogenic variants were detected in six probands from six unrelated families, exclusively clustered in the hereditary retinopathy cohort compared with those with other ocular conditions. Their phenotypes were difficult to distinguish from other forms of hereditary retinopathies, in whom three with *MSN* nonsense variants showed phenotypes mimicking FEVR, while the remaining three had performances resembling retinitis pigmentosa. Though similar to other forms of retinopathy, some difficult-to-notice features of these patients were observed, which may be a good point of discrimination. Specially, ocular involvement was the initial and only sign of the six patients, which was in contrast to previous reports in which retinopathy was usually the accompanying sign of systemic abnormalities of HID. Recognizing such retinopathy may be the initial and only presenting phenotype of systemic HID and may contribute to good clinical care of these patients (**Figure 5**).

Autoimmune retinopathy is the best-known form of ocular involvement in HID-related retinopathy caused by antibodies targeting the retina (8). Autoimmune polyendocrinopathy syndrome type I (APS1), as the systematic autoimmune disease firstly identified to be caused by variants of a single gene (43), is characterized by a clinical triad of chronic mucocutaneous candidiasis, hypoparathyroidism, and primary adrenal insufficiency (44). Autoimmune regulator (AIRE) protein is well known for its critical role in the immune tolerance process (43). It functions as a key regulator by facilitating the expression of tissue-specific antigens within the thymus and promoting the elimination of self-reactive T cells (45). Defects in this key regulator can lead to the breakdown of self-antigen expression and loss of function in establishing central tolerance to various autoantigens (46). Though the human eye exists in a special immune-privileged environment (47), it still could be attacked under the organ-specific autoimmune dysfunction background due to AIRE deficiency (48). The infiltration of CD4+ and CD8+ T cells along with an increased expression of proinflammatory cytokines on the ocular surface have been observed in mice experiments, which suggested immune-mediated mechanism function in the disruption of the ocular surface barrier (49, 50). The initial symptoms of APS1 are usually infection in childhood, followed by hypoparathyroidism by age 10 and adrenal insufficiency by age 15 (51–53). Keratoconjunctivitis has been long-established as associated with APS1 when the eye is involved (54), while a limited number of patients with APS1 have been found to present with autoimmune retinopathy (10, 35–40). A total of 12 reported patients in the literature exhibited with consistent retinal changes resembling retinitis pigmentosa, which showed a pale optic disc, attenuated retinal arterioles, and bone-spicule pigment changes in the periphery with retinal atrophic changes (36). Other examinations showed disruption of retinal structures on OCT and extinguished photoreceptor responses as recorded by ERG, but no other features of the retina different from retinitis pigmentosa were found. Prior to the identification of autoimmune retinopathy, 11 of the 12 patients have been reported to present with systemic symptoms. Only one youngster presented initially with ocular symptoms. Rapid and painless vision loss that was as severe as no light perception and extinguished both rod and cone responses suggested a diagnosis of Leber’s congenital amaurosis. No positive genetic causes were identified when focus on the genes related to Leber’s congenital amaurosis. It was not until the occurrence of autoimmune hepatitis at the age of 3 that autoimmune-related disorders were suspected and confirmed by genetic findings (10). The patient identified in our cohort exhibited widespread tapetoretinal degeneration without obvious pigment deposits and hyper-autofluorescence change along the major vascular arcade on the FAF, suggesting an autoimmune nature (55). However, no frequent candida infection and neither signs nor clinical examination abnormalities of hypoparathyroidism or adrenal insufficiency were noted upon follow-up of this patient. It is suggested that systemic disorders should be taken into consideration when no ophthalmic genetic causes can be identified in patients exhibiting ocular symptoms resembling other types of retinopathies and in the absence of systemic manifestations. As the most important human sense, the initial detection of autoimmune retinopathy in APS1 through routine ophthalmic examinations may be an early warning sign before systemic abnormalities occur, similar to the early diagnosis of retinopathy in *CEP290*-associated Senior–Loken syndrome, thus providing a window of time to treat a kidney disease (56).

Starting with the case of *AIRE*-associated autoimmune retinopathy, it makes us wonder if there are more patients with hereditary retinopathies in our cohort for whom no ophthalmic relevant genetic factors have been identified that might be attributed to variants in HID-related genes. In addition to autoimmune retinopathy, other retinal dystrophic changes have been reported as ocular manifestations of HID. So, our study included not only genes related to autoimmune retinopathy but also others associated with HID-related retinal degenerative changes. Previously, a heterozygous germline variant in *CBL* that was involved in the RAS-MAPK signaling pathway has been reported to cause Noonan syndrome-like phenotype with a predisposition to malignancies (57), while somatic mutations in *CBL* have been found to lead to myeloproliferative disorders including juvenile myeloid leukemia (JMML) (58). The CBL protein exhibiting ubiquitin ligase (E3) activity has been identified as a significant negative regulator in the RAS-MAPK transduction cascade (59), a pivotal process in normal cellular mediating processes, by promoting the internalization and retrogradation of the epidermal growth factor receptor protein (EGFR) (60). The ubiquitination of E3 protein plays a crucial role in the regulation of the immune system, encompassing both innate and adaptive responses (61). Maintaining the balance between activation and inhibitory signals, the CBL protein contributes significantly to immune homeostasis (62–64). It has been demonstrated that pathogenic germline variants in the *CBL* gene impact the ubiquitylation and migration of EGFR, resulting in loss of downregulation function of the CBL protein in the RAS-MAPK pathway, which leads to Noonan syndrome and other RASopathies (65). Based on the Cbl knock-out mice model, it has been observed that autoimmune disorders were predisposed to happen, highlighting the critical role of CBL in immune regulation. Furthermore, potential therapy targeting the ubiquitin ligase has been reported to be a novel approach to autoimmunity disease (66). Only one published study described five patients with *CBL* variants who had various retinal performances, including two with peripheral pigmentary changes mimicking retinitis pigmentosa, two with optic dystrophy, and one with retinal detachment due to recurrent uveitis, four of whom had chronic hepatosplenomegaly for a long time and one who had idiopathic thrombocytopenic purpura since childhood before visual loss (12). Our study, for the first time, reported a patient with a characteristic retinal manifestation as the initial and only sign of a pathogenic germline variant in *CBL*. Pigmentary disturbance of the macula with sightly retinal tapetoretinal degeneration as observed with an ophthalmoscope indicated a diagnosis of retinitis pigmentosa, while special rhagadiforme-like degeneration with a hyper-autofluorescence change found in the far-peripheral retina attracted our attention. Such rhagadiforme-like degeneration was extremely rare and different from other inherited retinal degenerations commonly seen in our clinic. A genetic test was suggested, and a pathogenic germline mutation in *CBL* was identified. It was suggested that, in addition to OCT and ERG examinations, wide-field fundus examination as well as autofluorescence photography were also valuable in the initial determination of the autoimmune characteristics of retinal degeneration. Retinal degeneration associated with HID may present with hyper-autofluorescent performance which was distinct from retinitis pigmentation with a hypo-autofluorescent change (55).

Of the 12 genes related to retinal vasculature inflammation or posterior uveitis, only three hemizygous nonsense variants in *MSN* were identified in our cohort. Moesin (*MSN*) is one of the ezrin–radixin–moesin (ERM) family member proteins involved in the function of actin cytoskeleton by jointing cortical actin filaments to the plasma membrane proteins, which plays an essential role in the normal immune response by regulating lymphocyte activation and migration (67). Deficiency or overexpression of ERM-mediated interconnection in a mouse model has been found to be associated with disrupted lymphocyte homing (68). Highly expressed in lymphocytes, moesin determines the formation of the immunological synapse in B and T cells and controls the proliferation of CD8+ and CD4+ Tregs, whose deficiency has been found to result in persistent lymphopenia in the peripheral blood (69). Previously, variants in *MSN* have been reported to cause a novel form of X-linked recessive primary immunodeficiency (IMD50) that is characterized with recurrent infections in multiple organs (24). To the best of our knowledge, a total of 14 patients have been reported to have *MSN*-associated IMD50, including 11 probands carrying variant c.511C>T/p.(R171W), which results in mRNA degradation followed with protein expression reduction, and three patients were detected with nonsense variants (24, 42, 69–71). One boy patient had X-linked inflammatory bowel-like disease that was reported to be caused by nonsense variant in *MSN* (72). Moreover, moesin has been found to involve vascular endothelial dysfunction and to be associated with autoimmune-related vasculitis (73, 74). To date, only one reported patient with *MSN* variant has been described with ocular involvement and who had posterior exudative retinal detachment caused by chronic inflammation (42). In our study, no patients with the p.(R171W) variant or other likely pathogenic missense in *MSN* were identified. All three hemizygous nonsense *MSN* variants were clustered in patients with exudative vitreoretinopathy resembling the FEVR phenotype. Differently from other forms of FEVR, wide-field special tapetoretinal degeneration was observed in our patients. All three patients had no complaints of other systemic infections at the time of presentation, except for eye disease. In the situation that systemic symptoms were absent, a diagnosis of FEVR was likely to be carried out. However, no pathogenic or likely pathogenic variants in genes known to be the cause of FEVR were identified. For the first time, our study proposed that sight-threating exudative vitreoretinopathy phenotype was a characteristic ocular manifestation of *MSN*-associated autoimmune disease, which may be the initial and only signs. We are eager to further confirm our findings in more cases. Recognizing the correlation between exudative vitreoretinopathy and *MSN* is valuable, reminding us that such systemic HID-related exudative vitreoretinopathy should be recalled in the clinical care of these patients without obvious systemic abnormalities and identified genetic causes to explain the FEVR-like phenotypes. Moreover, patients with *MSN*-associated IMD50 should be aware of ocular disease to prevent severe sight loss due to retinal detachment.

The nuclear factor kappa B (NF-κB) signaling cascade is a widely recognized and crucial pathway that triggers immune cell activation and tightly linked to the transcription of pro-inflammatory and cell survival genes (75). Both Blau syndrome and Haploinsufficiency A20 are auto-inflammatory diseases resulting from the excessive activation of the NF-κB pro-inflammatory pathway. Blau syndrome was caused by gain-of-function variants in *NOD2* gene, which lead to an aberrant auto-activation of the NF-κB pathway (21). In contrast to the understanding that loss-of-function variants of *NOD2* were considered nonpathogenic for Blau syndrome (21), Haploinsufficiency A20 is attributed to loss-of-function variants in *TNFAIP3* gene (22). The A20 protein encoded by *TNFAIP3* has been reported to play an essential role in the NF-κB pathway by regulating its signals through a deubiquitinating activity. Loss-of-function variants in *TNFAIP3* result in the inadequate suppression of the NF-κB signaling, consequently leading to inflammation (76). As rare autosomal dominant auto-inflammatory diseases, both Blau syndrome and Haploinsufficiency A20 are characterized by early-onset systemic inflammation as well as ocular inflammation manifestations. The classical clinical triad of Blau syndrome includes granulomatous uveitis, arthritis, and skin rashes (77, 78), whereas Haploinsufficiency A20 was presented with non-granulomatous uveitis, skin rashes, and oral or genital ulcers (22). Despite the hallmark manifestation of the two diseases that has been recognized, the diagnosis remains challenging because of their variable phenotype, which commonly overlaps with other inflammatory conditions (78). Recent advances in the treatment of the two autoinflammatory diseases have underscored the critical importance of the early identification of these patients, as it can significantly improve their prognosis. To achieve this goal, accurate and efficient diagnosis is necessary, which requires the detection of a pathogenic variant in *NOD2* or *TNFAIP3* (22, 79–81).

Non-infection ocular inflammation related to other systemic autoimmune disorders, such as anterior uveitis associated with ankylosing spondylitis, Vogt–Koyanagi–Harada disease, and Behçet’s disease (82), usually presents with significant symptoms which are predominantly inflammatory-related, including severe photophobia and pain, obvious ciliary congestion, anterior chamber flare, keratic precipitates, iris nodule, and so on. The patients with these obvious symptoms and signs suggestive of uveitis usually directly visit the uveitis clinic of ours rather than our genetic eye clinic. Therefore, our cohort mainly consists of patients with suspected hereditary ocular diseases, often presenting with ocular manifestations difficult to detect and which are unexplained. However, the feature of our cohort was also our limitations, namely, that the exact proportion of uveitis is not known. Another limitation of our study was the lack of a long-term follow-up of systemic symptoms in these patients, which we aspire to perform in the future.

Although our research mainly focused on a large cohort comprising thousands of subjects with hereditary ocular diseases, our findings should be applicable for validation in other larger cohorts of diverse individuals. The ocular phenotypes observed in both our registered patients and reported cases in previous literature could be explained by the identified variants with explicit pathogenicity. Pathogenic variants in HID-related genes exclusively clustered in the hereditary retinopathy cohort as compared to the cohort with other ocular abnormalities and the general population. Moreover, variants in genes attributed to other forms of inherited eye diseases have been excluded at first. Based on our findings and a comprehensive review of the literature, the conclusion that individuals detected with pathogenic variants in one of the 20 genes related to HID might initially or exclusively present with ocular degenerative or proliferative mimicking retinitis pigmentosa or FEVR could be drawn. Of course, it should be emphasized that potential variants in other known genes related to hereditary ocular diseases should be excluded, and systemic immunological laboratory indicators should be given careful attention. Also, we are eager to carry out in the future a larger population study not limited to specific patients with ocular abnormalities to further validate our findings. Furthermore, when noticing specific manifestations of the retina, including degenerative, proliferative, inflammatory, and vascular changes, consideration should be given to the possibility of HID diagnosis.

In summary, our study, for the first time, reported six patients with retinopathy as the initial and only sign of HID at the time of visit, in contrast to previous studies that reported patients with retinopathy as a concomitant sign of systemic abnormalities. Our findings are a good reminder that such retinal features should be considered as possibly HID-related even though no systemic clinical features are present. In patients with degenerative changes mimicking retinitis pigmentosa and vascular changes resembling FEVR for which no genetic cause can be found, attention should be paid to the importance of HID-related retinopathy. Earlier recognition of such an ocular phenotype of HID may provide a valuable time window for tracking and caring for potential and serious to systemic symptoms of these patients.

## Data availability statement

The datasets presented in this study can be found from the following link: https://bigd.big.ac.cn/gsa-human/browse/HRA005029. The accession number is HRA005029.

## Ethics statement

The studies involving humans were approved by the institutional review board of Zhongshan Ophthalmic Center. The studies were conducted in accordance with the local legislation and institutional requirements. Written informed consent for participation in this study was provided by the participants’ legal guardians/next of kin.

## Author contributions

YW, YJ, JW, XX, SL, XJ, WS, and QZ recruited the individuals diagnosed with different forms of ocular conditions and collected the clinical records. SL, XX, and XJ prepared the genomic DNA from venous blood. XX, SL, PW, and QZ performed whole-exome analysis and targeted exome sequencing. YW, PW, and QZ participated in the bioinformatics analysis of exome sequencing data and in the review and classification of clinical data. YW performed Sanger sequencing. QZ designed the study. YW wrote the manuscript. QZ critically revised the manuscript. All authors contributed to the article and approved the submitted version.
